# Sorafenib-induced Facial Acneiform Eruption

**DOI:** 10.7759/cureus.4545

**Published:** 2019-04-26

**Authors:** Maria C Mijares, Adam S Aldahan, Hector H Gonzalez, Juan P Jaimes

**Affiliations:** 1 Internal Medicine, Florida Atlantic University Charles E. Schmidt College of Medicine, Boca Raton, USA; 2 Dermatology, University of Minnesota, Minneapolis, USA

**Keywords:** sorafenib, acneiform eruption, acne, cutaneous adverse event

## Abstract

Sorafenib is a multikinase inhibitor that is used to treat hepatocellular carcinoma by inhibiting tumor cell growth and angiogenesis. Cutaneous adverse events of sorafenib are commonly reported, with alopecia and hand-foot skin reactions most frequently encountered. Acneiform eruptions represent rare adverse events that have only been reported at high doses of sorafenib. We present a patient who started low dose sorafenib for hepatocellular carcinoma and subsequently developed a fulminant facial acneiform eruption in the absence of other cutaneous adverse events. Treatment included topical clindamycin and tretinoin with some improvement. Facial acneiform eruption represents a rare consequence of sorafenib that has not previously been described at low doses. Additionally, acneiform papules in the absence of other cutaneous adverse events is unusual. The cutaneous mechanism is not well understood but may be related to indirect epidermal growth factor receptor inhibition or direct cytotoxic effects on eccrine glands. Topical treatment produces only minimal improvement in patients who continue sorafenib therapy. Discontinuation of the drug is usually unwarranted except in special circumstances.

## Introduction

Sorafenib is a multikinase inhibitor that treats solid organ tumors through inhibitions of cell growth and angiogenesis. Cutaneous adverse effects such as alopecia and hand-foot skin reactions have been well established. Only a handful of cases of acneiform papules have been associated with high dose sorafenib. Herein, we present a case of facial acneiform eruption in the absence of other skin manifestations following treatment with low dose sorafenib.

## Case presentation

A 68-year-old African American male presented to an outpatient dermatology clinic in November 2018 for evaluation of numerous skin-colored papules on his face. The patient had a history of hepatitis C that was successfully treated in November 2015. He was diagnosed with hepatocellular carcinoma in April 2016 and underwent laparoscopic ablation in May 2016. Routine imaging did not show any progression of disease for approximately two years. In October 2017, he was diagnosed with a gastrointestinal stromal tumor. He was started on imatinib 400 milligram (mg) daily in April 2018 and did not experience any cutaneous side effects.

Despite prior negative liver imaging after his ablation, routine magnetic resonance imaging in July 2018 revealed new liver masses. In August 2018, a liver biopsy confirmed well differentiated hepatocellular carcinoma. In September 2018, the patient was started on sorafenib at a palliative dose of 200 mg twice daily. During this time, he continued imatinib therapy.

Approximately two weeks after initiating sorafenib, he developed a sudden facial papular eruption. The papules were nonpruritic and nontender with no associated photosensitivity. He denied a history of similar papular eruptions in the past. Aside from sorafenib, there were no new medications, supplements, or hazardous exposures. He denied other cutaneous manifestations such as hair loss, palmar erythema, or desquamation.

**Figure 1:**
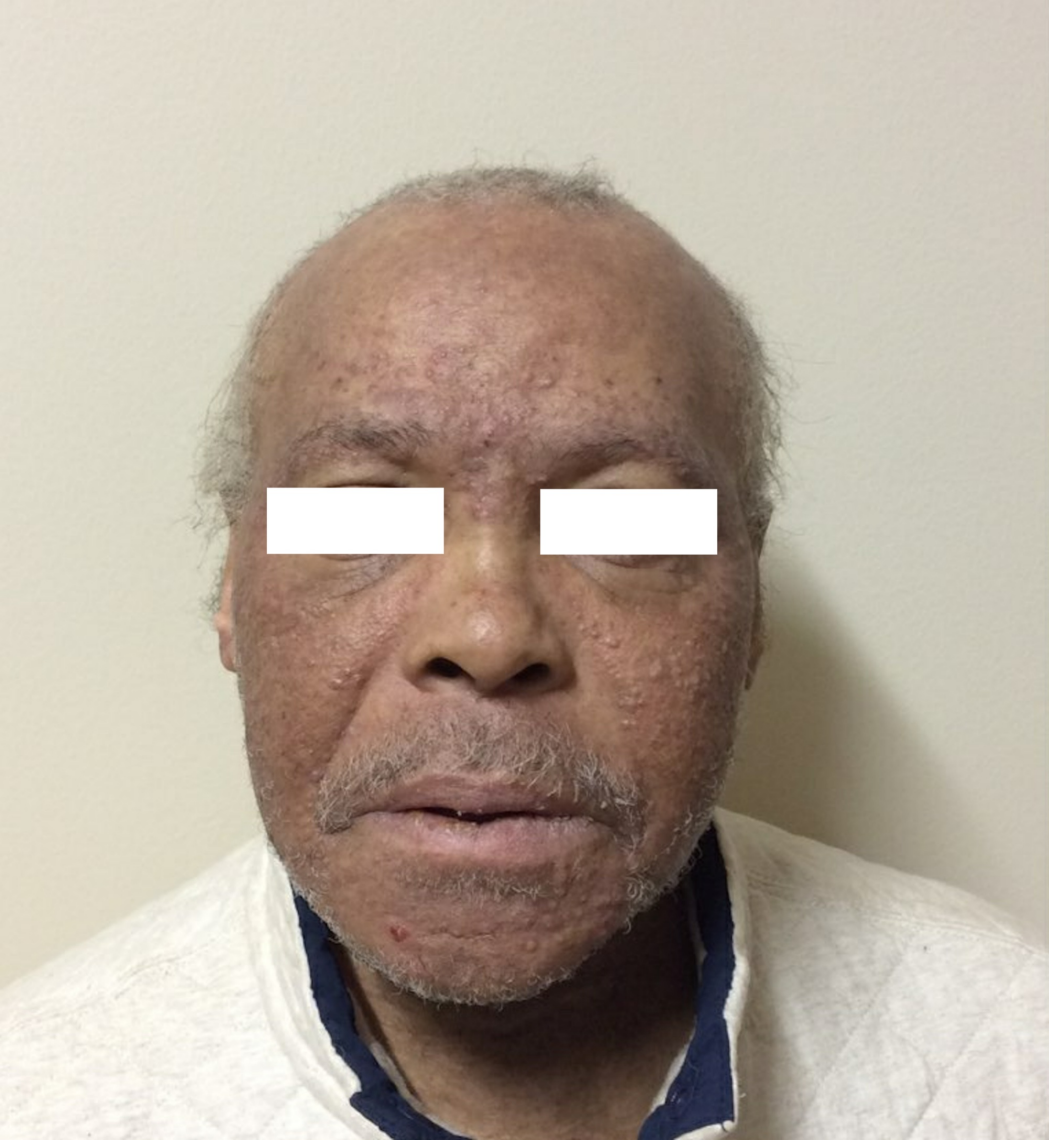
Monomorphic skin-colored to erythematous comedonal papules diffusely present on the forehead, glabella, cheeks, root of the helix and chin. The upper and lower eyelids and upper cutaneous lip were spared.

**Figure 2:**
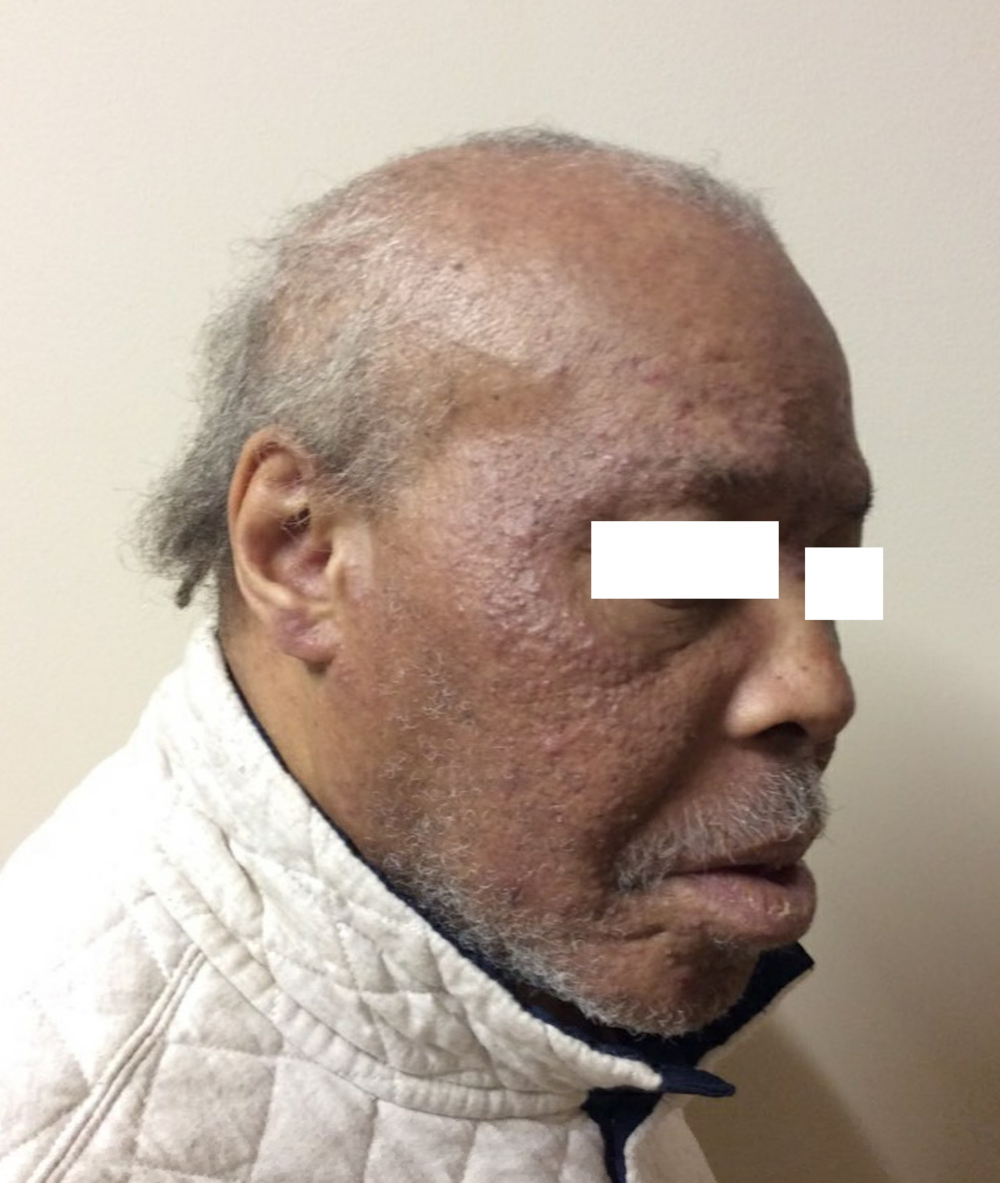
Extension of monomorphic skin-colored to erythematous comedonal papules to earlobes.

Dermatologic examination revealed monomorphic skin-colored to erythematous comedonal papules diffusely present on the forehead, glabella, nose, cheeks, root of the helix and chin (Figures 1, 2). There were a few inflammatory papules identified. The upper and lower eyelids and upper cutaneous lips spared. There were no inflammatory papules or pustules identified. There was no drainage, bleeding, erosion, or crust. There were no lesions on the trunk or extremities, and the acral surfaces were unaffected.

Given the temporal relationship between sorafenib initiation and the facial acneiform eruption, sorafenib was implicated as the causal factor. This cutaneous adverse event was not severe enough to warrant medication discontinuation. The patient was prescribed tretinoin cream and clindamycin gel. After two months of topical treatment, he had modest improvement of his cheeks and chin but no reduction in the forehead lesions.

## Discussion

Sorafenib is a multikinase inhibitor used for the treatment of advanced solid cancers including hepatocellular, thyroid, and renal cell [1]. It works by inhibiting kinase activity of proto-oncogene, serine/threonine kinase (C-RAF and B-RAF). Sorafenib targets the intracellular adenosine triphosphate (ATP) binding domain of tyrosine kinase, preventing phosphorylation and downstream signaling of factors such as vascular endothelial growth factor receptor, platelet derived growth factor receptor, fibroblast growth factor receptor, and epidermal growth factor receptor (EGFR) [2]. It is thought to halt tumor cell angiogenesis and proliferation through these mechanisms.

Various cutaneous adverse events have been reported with sorafenib. The most common include hand-foot skin reaction, facial and scalp eruptions, alopecia, and pruritis [3]. Hand-foot skin reaction is the most taxing cutaneous manifestation, characterized by tender erythematous hyperkeratotic lesions on the palms, soles, and weight bearing sites [4]. This is distinct from the hand-foot syndrome seen in other chemotherapeutic agents, which presents as symmetric desquamative acral erythema. Other less common adverse events of sorafenib include subungual splinter hemorrhages, erythema multiforme, and keratoacanthomas [5,6].

Sorafenib-induced acneiform eruption is a very rare adverse event that is not well described in the literature. The few reported cases were only seen with high doses, and there appears to be a dose dependent relationship. One case described complete resolution in acneiform lesions after decreasing the sorafenib dose from 800 mg twice a day to 400 mg twice a day [7]. Our patient developed acneiform lesions while on a low dose of 200 mg twice a day, although he had also been on imatinib at the time. Imatinib is another tyrosine kinase inhibitor that has been associated with acne eruptions [8,9]. Although our patient had not developed cutaneous side effects on imatinib alone, it is plausible that adding low dose sorafenib may have reached a certain threshold to cause the acneiform eruption. This still does not explain the lack of other cutaneous adverse events more commonly associated with tyrosine kinase inhibitors.

The mechanism of sorafenib-induced cutaneous adverse reaction is not well understood. Free radical production has been entertained as a possible cause, although one study demonstrated that sorafenib actually produces antioxidants in vivo [10]. Involvement of palms and soles may be caused by direct toxic effect on eccrine glands [11]. In contrast to tyrosine kinase inhibitors, EGFR inhibitors are well known to cause acneiform eruptions [12]. Given the indirect downstream effects of sorafenib on EGFR, the mechanism of acneiform lesion production may be somehow related to EGFR inhibition. This may also explain the apparent dose dependence of acneiform lesion development. More research is necessary to further understand these mechanisms.

Treatment options include topical retinoids, antibiotics, and benzoyl peroxide, although there is expected to be minimal improvement without removing the offending agent. In most cases the cutaneous symptoms do not warrant discontinuation of sorafenib; however, alternative therapy should be considered in cases that are disfiguring or significantly reduce quality of life [7].

## Conclusions

Facial acneiform eruption is a rare cutaneous adverse effect of sorafenib. The effect seems to be dose dependent but may also develop in low doses when used alongside other similarly acting agents. Although clearance of these lesions is unlikely with continued sorafenib exposure, discontinuation of sorafenib is usually not necessary.
